# Role of PI3-Kinase in Angiotensin II-Induced Cardiac Hypertrophy: Class I Versus Class III

**DOI:** 10.3389/fphar.2021.608523

**Published:** 2021-02-16

**Authors:** Tiecheng Zhong, Zonggui Wang, Sayeman Islam Niloy, Yue Shen, Stephen T. O’Rourke, Chengwen Sun

**Affiliations:** ^1^ Department of Pharmaceutical Sciences, North Dakota State University, Fargo, ND, United States; ^2^ Department of Otolaryngology, The Second Hospital, Jilin University, Changchun, China; ^3^ Institute of Pharmacology and Toxicology, Zhejiang Province Key Laboratory of Anti-Cancer Drug Research, College of Pharmaceutical Sciences, Zhejiang University, Hangzhou, China

**Keywords:** PI3-kinases, cardiac hypertrophy, heart failure, angiotensin II, autophagy

## Abstract

Cardiac hypertrophy is an adaptive response to cardiac overload initially but turns into a decompensated condition chronically, leading to heart failure and sudden cardiac death. The molecular mechanisms involved in cardiac hypertrophy and the signaling pathways that contribute to the switch from compensation to decompensation are not fully clear. The aim of the current study was to examine the role of PI3-kinases Class I (PI3KC1) and Class III (PI3KC3) in angiotensin (Ang) II-induced cardiac hypertrophy. The results demonstrate that treatment of cardiomyocytes with Ang II caused dose-dependent increases in autophagy, with an increasing phase followed by a decreasing phase. Ang II-induced autophagic increases were potentiated by inhibition of PI3KC1 with LY294002, but were impaired by inhibition of PI3KC3 with 3-methyladenine (3-MA). In addition, blockade of PI3KC1 significantly attenuated Ang II-induced ROS production and cardiomyocyte hypertrophy. In contrast, blockade of PI3KC3 potentiated Ang II-induced ROS production and cardiac hypertrophy. Moreover, blockade of PI3KC1 by overexpression of dominant negative p85 subunit of PI3KC1 significantly attenuated Ang II-induced cardiac hypertrophy in normotensive rats. Taken together, these results demonstrate that both PI3KC1 and PI3KC3 are involved in Ang II-induced cardiac hypertrophy by different mechanisms. Activation of PI3KC1 impairs autophagy activity, leading to accumulation of mitochondrial ROS, and, hence, cardiac hypertrophy. In contrast, activation of PI3KC3 improves autophagy activity, thereby reducing mitochondrial ROS and leads to a protective effect on Ang II-induced cardiac hypertrophy.

## Introduction

Cardiac hypertrophy refers to the enlargement of cardiomyocytes with a series of physiological and pathological modifications such as heart expansion, altered gene expression, enhanced protein synthesis, and contractile machinery reorganization (Harvey and Leinwand, 2011). Cardiac hypertrophy is initially adaptive to compensate for the increasing physiological oxygen and nutrient demands during exercise or pregnancy (Hill and Olson, 2008). Pathological state-related stresses such as hypertension, obesity, and myocardial infarction elicit maladaptive cardiac hypertrophy that is associated with unusual cardiac structures and metabolism, as well as abnormal cardiac functions (Hill and Olson, 2008). If improperly treated, cardiac hypertrophy is a detrimental factor that would ultimately result in heart failure and sudden cardiac death, threatening the well-being of individuals and creating a tremendous burden on society as a whole.

Among those physiological and pathological factors that cause cardiac hypertrophy, Angiotensin II (Ang II) stimulation plays a pivotal role (Sadoshima et al., 1993). Ang II acts as a vasoconstrictor, thus increasing blood pressure and cardiac load; it also stimulates cardiomyocytes directly via activation of angiotensin receptor type I receptors (AT1R) (Hunyady and Catt, 2006). PI3-kinases are involved in the intracellular downstream signal-transduction pathways of AT1 receptors in cardiomyocytes (Wenzel et al., 2006). Two classes of PI3-kinase, Class I (PI3KC1) and Class III (PI3KC3), have been identified in the heart (Misawa et al., 1998). Targeted over-expression of PI3KC1 increased heart size and manifested cardiac hypertrophy. Conversely, dominant negative (DN) treatment-induced inactivation of certain essential signal-transduction components, such as the p110 subunit of PI3KC1 in the heart, attenuated cardiac hypertrophy in transgenic mice (Shioi et al., 2000). However, the role of PI3KC1 vs. PI3KC3 in Ang II-induced cardiac hypertrophy is still unknown.

Previous studies demonstrate that reactive oxygen species (ROS) derived from NADPH oxidases are involved in Ang II-induced cardiac hypertrophy through a PI3-kinase-dependent pathway (Yao et al., 2008). However, genetic modification resulting in inactivation of the gp91phox subunit, a vital component within NADPH oxidase, in transgenic mice did not show significant benefits to alleviate Ang II-induced hypertension or cardiac hypertrophy (Touyz et al., 2005). These findings suggest there might be an alternative intracellular pathway to generate ROS in cardiomyocytes under conditions of prolonged Ang II exposure. Recent studies demonstrate that damaged mitochondria may also generate ROS in cardiomyocytes (Pei et al., 2016). However, the intracellular mechanisms involved in accumulation of damaged mitochondria-derived ROS in Ang II-induced cardiac hypertrophy are still unclear.

It is well known that autophagy is a very important intracellular process to protect cells from hazardous material accumulation by scavenging damaged mitochondria or proteins that produce reactive oxygen species (ROS). Accumulating evidence indicates the possible involvement of autophagy in the pathophysiology of Ang II-induced cardiac hypertrophy (Zhou et al., 2016). Thus, the major aim of the current study was to test the hypothesis that activation of PI3-kinases is involved in Ang II-induced cardiac hypertrophy by regulating autophagy activity and mitochondrial ROS generation.

## Methods

### Animals

Twelve-week-old male Sprague-Dawley (SD) rats were used in this study (purchased from Charles River Laboratories International, Wilmington, MA). Rats were housed at 25 ± 2°C on a 12:12-h light-dark cycle and provided with food and water *ad libitum*. All animal protocols were approved by the North Dakota State University Institutional Animal Care and Use Committee and the Jilin University Institutional Animal Care and Use Committee (IACUC). All experiments were carried out in accordance with guidelines and regulations approved by the IACUCs.

### *In vivo* Myocardial Gene Delivery

Construction and titration of lentiviral vectors of Lv-GFP (negative control) and Lv-DNp85 (the dominant negative p85α subunit of PI3KC1) were performed as described in our previous publication (Sun et al., 2009). The lentiviral vectors were transferred into the myocardium by injection into the root of the aorta, as published previously (Iwanaga et al., 2004; Yan et al., 2015). Briefly, male adult SD rats were anesthetized with an O_2_ (1 L/min) and isoflurane (3%) mixture administrated through a nose cone. Left anterior thoracotomy was carried out in the left second intercostal space. For arterial occlusion, ligatures were loosely looped around the main pulmonary arteries and the ascending aorta. To inject viral particles into the coronary arteries, a vascular catheter was inserted through the right carotid artery into the aortic root between the aortic valve and the occlusion ligature loop. Ice packs were used to create a general hypothermic environment for the rats in order to cool their body temperatures below 26°C. Then, a protective cardioplegic solution (2 μL/g body weight) was injected, containing (in mM): NaCl 110, KCl 20, MgCl_2_ 16, NaHCO_3_ 10, and CaCl_2_ 1.2 via the arterial catheter, followed by injection of Lv-GFP or Lv-DNp85 viral particles (200 μL 2×10^10^ TU/mL). Substance P was also added to the cardioplegic solution, at a final concentration of 25 μg/ml, to enhance permeability of the coronary artery wall and guarantee access of viral vectors into the myocardium. After injection, both occlusions were loosened and rat body temperature was heated back to normal using a heating pad. To enhance the gene transfection, Lv-DNp85 or Lv-GFP were also injected directly into the anterior ventricular well. The chest was then closed; intrathoracic air was evacuated by suction with a syringe.

### Evaluation of Heart Morphology

The morphology of hearts was evaluated after the hearts were transversely sectioned. Heart sections were fixed in 10% formalin/PBS solution. The cardiomyocyte morphology and cellular dimension were examined by hematoxylin and eosin (H&E) staining in cardiac sections (4∼5 µm thickness). The stained cardiac sections were visualized under light microscopy (Olympus). The cross-sectional diameter of single myocytes was measured by the cellular diameter crossing the nuclei, using Infinity Capture and Analyze Software under a microscope (Olympus). The outline of 100–200 cardiomyocytes was traced from each rat.

### Preparation of Primary Cardiomyocyte Culture

Primary cardiomyocyte cultures were prepared as described previously (Yao et al., 2010; Zhao et al., 2015). Briefly, 1-day-old neonatal SD rats were euthanized by overdose with sodium pentobarbital (200 mg/kg, *i. p.*, Sigma, St. Louis, MO). Ventricles of the heart were quickly excised, minced into small pieces in cold HBSS, and washed several additional times. The minced tissue was digested with 0.1% trypsin in a 37 °C water bath with shaking for 5-min rounds of tissue digestion (10–12 times). The supernatants from each incubation were pooled and added into an equal volume of DMEM containing 10% FBS. Isolated cells were then filtered with a 70 μm cell sieve and centrifuged (1,000 rpm) for 10 min. After centrifuge, supernatants were discarded and cell pellets were re-suspended in DMEM composed of 10% FBS and 1% penicillin-streptomycin. The cells were placed in an incubator for 1.5 h to allow non-myocyte cells, such as fibroblasts, to attach on the plate bottom. The cell suspension (final cellular density 5 × 10^5^ cells/cm^2^) was then transferred to a 24-well plate. In the first three days, 5-Bromodeoxyuridine (10^−4^ M) was added to suppress fibroblast growth. All of the manipulations were performed in a culture hood to ensure an aseptic environment. After the cell cultures reached confluence (5 days on average) in an incubator filled with a humidified atmosphere of 5% CO_2_ at 37°C, cardiomyocytes were used for *in vitro* experiments.

### Cardiomyocyte Morphology Examination and Autophagy Detection

Cultured cardiomyocytes were treated with the HBSS control, vehicle (0.1% DMSO) control, Ang II, LY-294002 (a PI3KC1 inhibitor), 3-MA (a PI3KC3 inhibitor), LY-294002 + Ang II, or 3-MA + Ang II for 24 h. Cardiomyocyte autophagy was determined using immunofluorescent staining with anti-MAP-LC3 antibodies as described in our previous publication (Yao et al., 2010). In brief, cells were fixed with 4% paraformaldehyde for 30 min. The cells were washed with fresh PBS containing 0.1% Triton X-100 three times. After pre-incubation with 3% bovine serum albumin (BSA) for 20 min, the cells were incubated with a mixture of primary antibodies against α-actin and LC3 (1:100 dilution) overnight at 4°C. After washing with PBS-Triton X-100 solution, cells were incubated with fluorescence-conjugated secondary antibodies (1:1,000 dilution) for 2 h at room temperature in the dark. The images were taken under a fluorescence microscope (Olympus). The cardiomyocytes were identified by α-actin positive cells. The puncta were counted inside of the cardiomyocytes that were identified with α-actin. The average number of puncta in each cell was calculated as described in previous literature (Hou et al., 2014). At least 10 different cells in each dish were randomly chosen to measure autophagy. A higher average number of puncta in each cell indicates a larger degree of autophagy occurence. The results are expressed as fold changes vs. HBSS control.

In addition, photographic images of cardiomyocytes taken with the fluorescence microscope were analyzed using computer software (Image Pro plus 6.0, Media Cybernetics, Bethesda, MD). The cell surface area of cardiomyocytes that were positively stained for sarcomeric actin was measured. At least 50 cells in each dish were randomly selected for surface area analysis.

### Measurement of ROS Production in Cardiomyocytes

Mitochondrial ROS production was determined using the superoxide-sensitive (O_2_^∙−^) fluorogenic probe MitoSox (Thermo Fisher, M-36008, Rockford, IL) as detailed in our previous publication (Guo et al., 2017). MitoSOX has a dihydroethidium (DHE) part linked to a triphenylphosphoninum (TPP) component and yields red fluorescence when oxidized (excitation/emission wavelength: 510/580 nm). This compound is more concentrated in the mitochondria than in the cytosol since the former has more positively charged TPP. Cultured cardiomyocytes were pretreated with the vehicle (DMSO, 0.1%) control, LY-294002, or 3-MA for 30 min. Then the cardiomyocytes were treated with Ang II (10^−6^ M) or HBSS control. Intracellular mitochondrial ROS levels were measured immediately after the addition of Ang II or HBSS control by incubation with MitoSOX (5 × 10^−6^ M, 15 min). The fluorescence resulting from intracellular probe oxidation was measured with a fluorescence microscope (Olympus) and analyzed with computer software (Image Pro plus 6.0).

### Western Blot Analysis

The protein levels of p-Akt and total Akt in heart tissue were assessed by Western Blots, as described in our previous publication (Yao et al., 2008). Briefly, antibodies against p-Akt or total Akt (Santa Cruz, Dallas, TX) were used as primary antibodies (1:500 dilution). Peroxidase-conjugated antibodies against rabbit IgG and mouse IgG (Bio-Rad, Hercules, CA) were used as secondary antibodies (1:15,000 dilution). Immunoreactivity was detected by enhanced chemiluminescence autoradiography. Films were analyzed using Image J.

### Data Analysis

All data are presented as means ± SE. Data were statistically analyzed using computer software (GraphPad Prism 5.0). Statistical significance was determined using one- or two-way ANOVA, as appropriate; and confirmed by either a Newman–Keuls or Bonferroni’s *post hoc* analysis. *p* values <0.05 were taken as significant. Significance levels are given in the text.

## Results

### Blockade of PI3KC1 Attenuated Ang II-Induced Cardiac Hypertrophy in Rats

**FIGURE 1:**
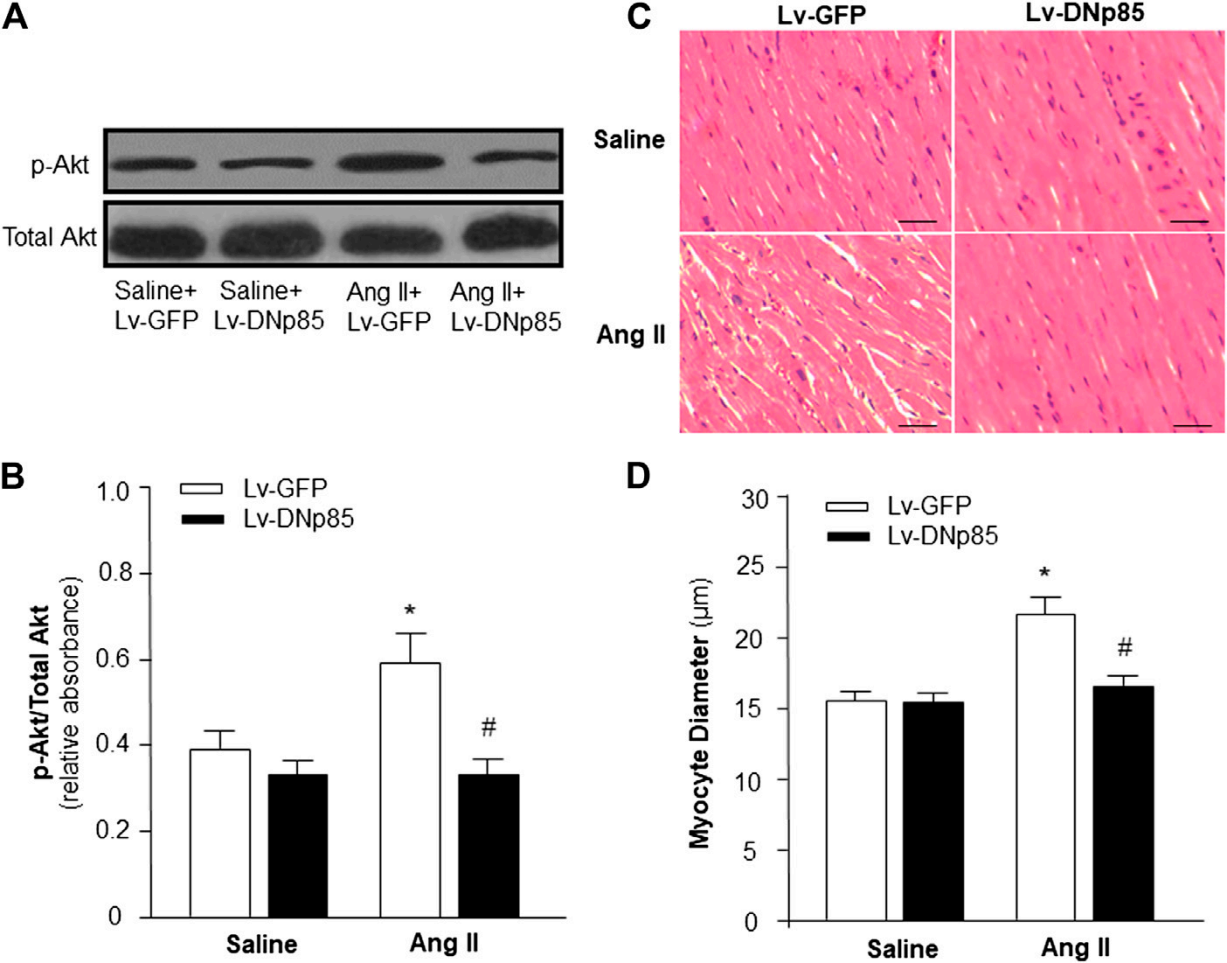
Effect of blockade of PI3KC1 on Ang II-induced cardiac hypertrophy in rat hearts. Cardiomyocyte diameter and Akt phosphorylation were examined in SD rats that received subcutaneous infusion of Ang II or saline control with cardiac transduction of Lv-DNp85 or Lv-GFP. **(A)** Representative western blots of phosphorylated Akt (p-Akt) and total Akt in the heart of each group of rats. **(B)** Bar graphs summarizing the ratio of phosphorylated Akt vs. total Akt. Data are presented as means ± SE (n = 6 rats). ^*^ *p* < 0.05 as compared with the group of saline + Lv-GFP. #*p* < 0.05 as compared with the group of Ang II + Lv-GFP. **(C)** Micrographs showing representative heart sections stained with hematoxylin/eosin in each group of rats. **(D)** Bar graphs summarizing diameter of cardiac myocytes from transverse cardiac sections of each group of rats. 100 cells per rat were observed randomly and averaged. Scale bar: 50 μm. Data are means ± SE (n = 6 rats in each group). *p < 0.05 vs. rats that received saline + Lv-GFP. #*p* < 0.05 vs. rats that received Ang II + Lv-GFP.

First, we determined the role of PI3KC1 in Ang II-induced cardiac hypertrophy by overexpressing the dominant negative class I PI3-kinase p85α subunit (DNp85) or GFP (control) using a viral vector-mediated gene-transfer technique in rat hearts through coronary arterial and cardiac injection. After injection, Ang II (200 ng/kg/min) or normal 0.9% saline were subcutaneously infused using osmotic pumps. After 4-weeks treatment with saline + Lv-GFP, saline + Lv-DNp85, Ang II + Lv-GFP, or Ang II + Lv-DNp85, the rats were euthanized and the cardiac tissue was collected. The PI3KC1 activity was detected using conventional Western Blots with antibodies against phosphorylated Akt (p-Akt) or total Akt. The results (Figures 1A,B) demonstrated that 4-weeks infusion of Ang II significantly increased the ratio of p-Akt vs. total Akt (0.38 ± 0.07 in the saline + Lv-GFP group vs. 0.59 ± 0.12 in the Ang II + Lv-GFP group, n = 6, *p* < 0.05), suggesting a chronic stimulatory effect of Ang II on PI3KC1 activity. Lentiviral vector-mediated overexpression of DNp85 dramatically attenuated Ang II infusion-induced phosphorylation of Akt (0.59 ± 0.12 in the Ang II + Lv-GFP group vs. 0.36 ± 0.06 in the Ang II + Lv-DNp85 group, n = 6, *p* < 0.05). The results demonstrate that chronic overexpression of Lv-DNp85 attenuates Ang II infusion-associated activation of PI3KC1.

The effects of Ang II infusion and Lv-DNp85 cardiac transduction on cardiac hypertrophy were evaluated in these four groups of rats by examining the morphology of cardiac myocytes in heart sections using H&E staining. The results are presented in Figures 1C,D, demonstrating that Ang II infusion significantly increased the diameter of cardiomyocytes, thus inducing severe cardiac hypertrophy (15.5 ± 0.5 μm in the Saline + Lv-GFP group vs. 22.5 ± 1.2 μm in the Ang II + Lv-GFP group, n = 6, *p* < 0.05). Ang II-induced increases in cardiomyocyte diameter were significantly attenuated by cardiac transduction of Lv-DNp85 (22.5 ± 1.2 μm in the Ang II + Lv-GFP group vs. 17.6 ± 1.1 μm in the Ang II + Lv-DNp85 group, n = 6, *p* < 0.05). The results demonstrate that blockade of PI3KC1 attenuates chronic Ang II infusion-associated cardiac hypertrophy.

### Effect of Ang II on Cardiomyocyte Autophagy

**FIGURE 2:**
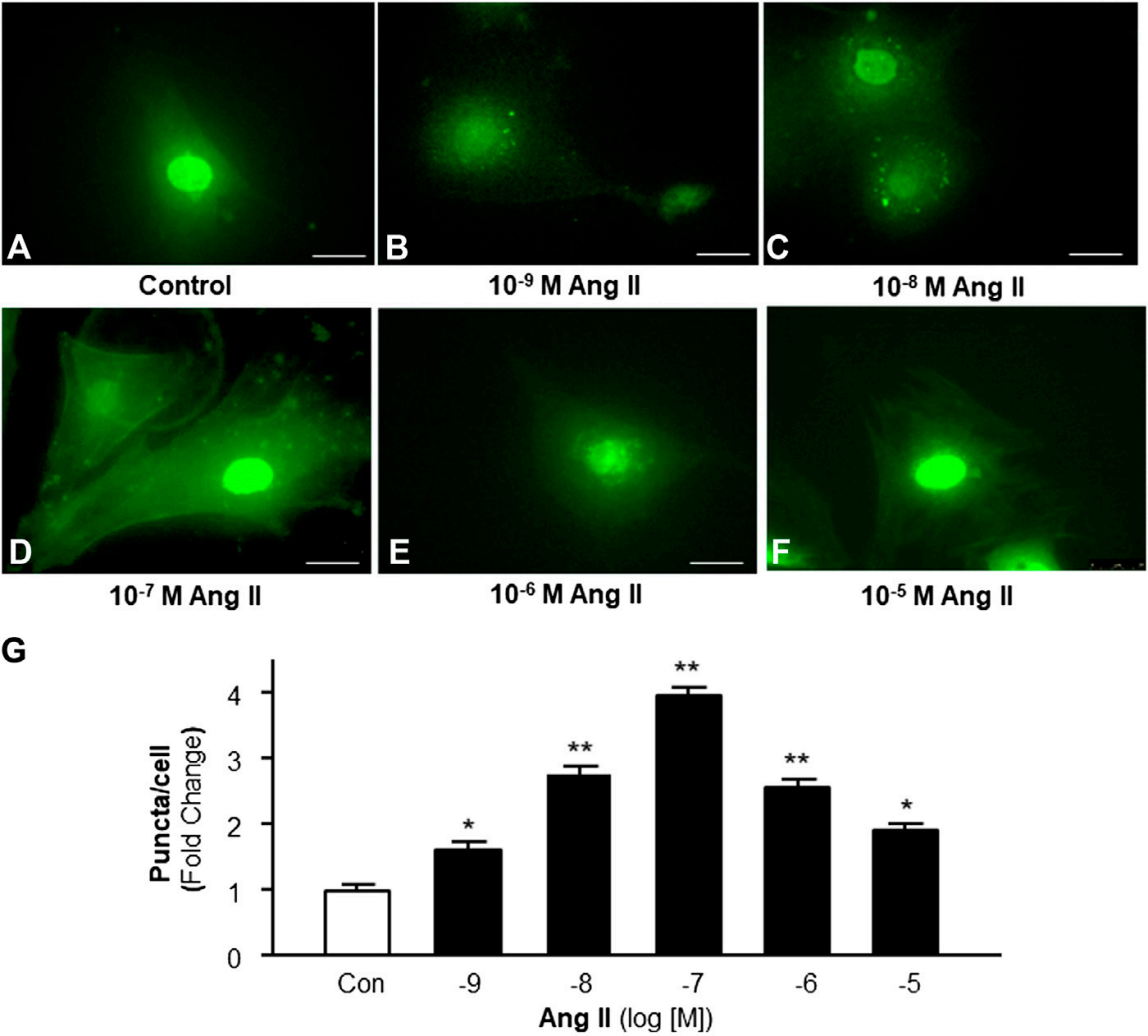
Effect of Ang II on autophagy in cardiomyocytes. Autophagy was detected with MAP-LC3 antibody immunofluorescence by counting intracellular puncta as formation of autophagosomes. **(A–F)** Representative fluorescence micrographs of cultured cardiomyocytes stained with anti-LC3 antibodies after treatments with HBSS control **(A)**, and Ang II at different concentrations (from 10^−9^ to 10^−5^ M, B-F). **(G)** Bar graphs showing the effect on autophagy under Ang II exposure at different concentrations. Data are mean ± SE, n = 10 in each group. *p < 0.05 vs. Control. **p < 0.01 vs. Control.

Accumulating evidence indicates the possible involvement of autophagy in the pathophysiology of Ang II-induced cardiac hypertrophy (Zhou et al., 2016; Guo et al., 2017). Autophagy is a very important intracellular mechanism to protect cells from hazardous material accumulation by scavenging damaged mitochondria or proteins that produce reactive oxygen species (ROS). Intracellular ROS plays an important role in the pathogenesis of cardiac hypertrophy (Sag et al., 2014). Thus, the effect of Ang II on autophagy in cardiomyocytes was examined using immunofluorescence staining with antibodies against microtubule-associated protein light chain 3 (MAP-LC3), an autophagosome marker. The autophagesomes within cardiomyocytes were visualized as green fluorescent puncta, which are able to be counted under a microscope. The results (as shown in Figure 2) demonstrate that Ang II treatment caused autophagic alterations with two phases: an increasing phase at lower dosages and a decreasing phase at high dosages.

### Effect of PI3KC1 and Ang II on Autophagy

**FIGURE 3:**
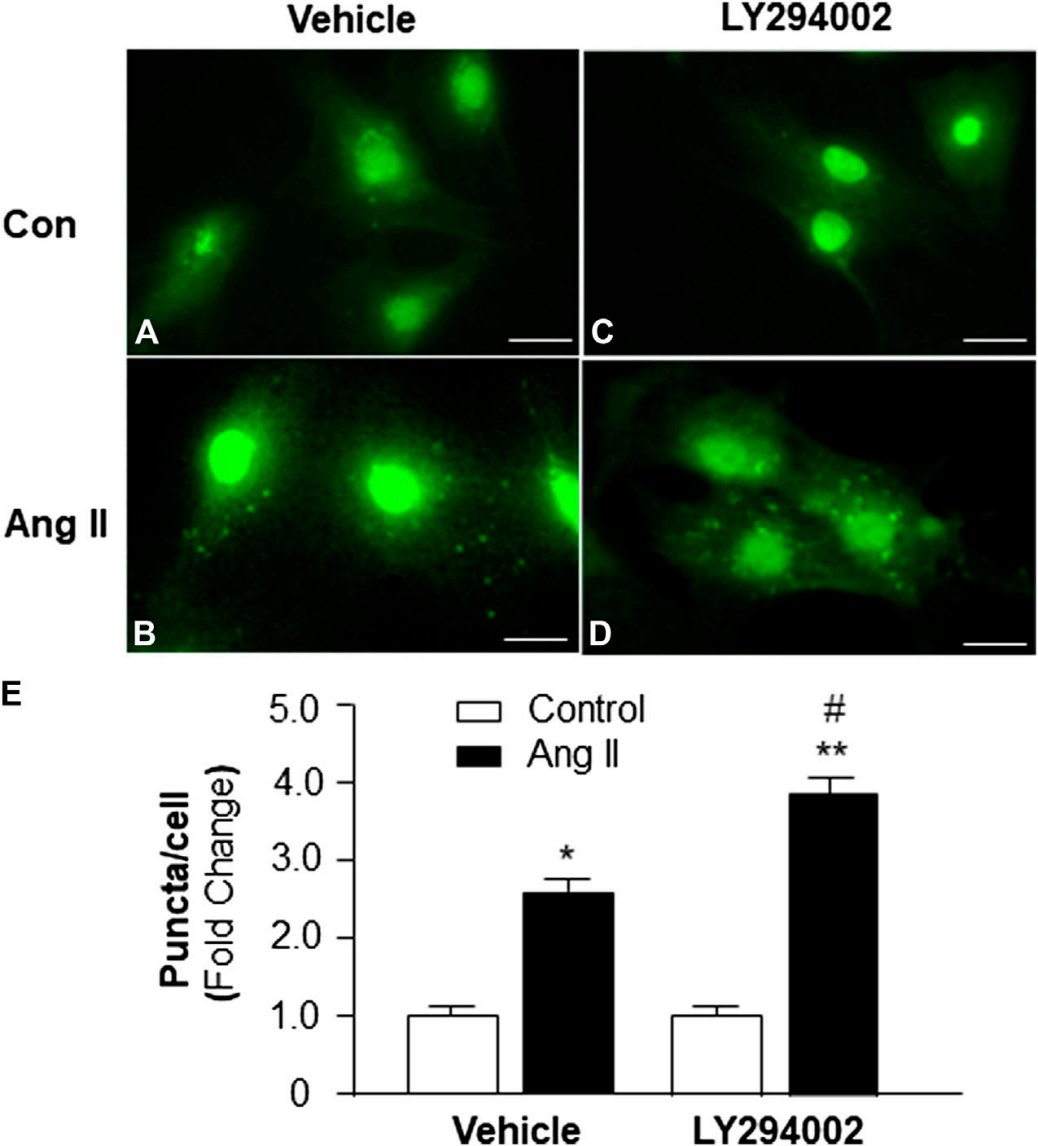
Effect of Ang II and blockade of PI3KC1 on autophagy in cardiomyocytes. Autophagy was examined in cardiomyocytes by immunostaining fluorescence with MAP-LC3 antibodies in cardiomyocytes treated with vehicle control, Ang II (10^−6^ M) with or without the PI3KC1 inhibitor, LY-294002 (10^−6^ M) for 24 h **(A–D)** Representative fluorescence micrographs of cultured cardiomyocytes stained with MAP-LC3 antibodies after treatments. **(E)** Bar graphs summarizing quantitative analysis of autophagic alterations in cardiomyocytes treated under the conditions described in the above. The scale in the images is 25 μm. Data are means ± SE, which were derived from three experiments and at least triplicate wells in each experiment.*p < 0.05 vs. cardiomyocytes that treated with vehicle control. **p < 0.01 vs. cardiomyocytes that treated with vehicle control. #*p* < 0.05 vs. cardiomyocytes that treated with Ang II.

To identify the role of PI3KC1 in Ang II-induced autophagy, autophagy was examined in cardiomyocytes treated with vehicle control or Ang II (10^−6^ M) with or without the presence of LY-294002, a PI3KC1 inhibitor. The results (as shown in Figure 3) demonstrate that Ang II (10^−6^ M) induced a significant elevation in autophagy in the presence of vehicle (DMSO, 0.1%, 1.00 ± 0.05 in control vs. 2.61 ± 0.12 in Ang II treatment, n = 3 experiments, *p* < 0.05). Treatment of cardiomyocytes with LY-294002 (10^−6^ M) significantly potentiated Ang II-induced autophagy by 46.4% (1.06 ± 0.07 in LY-294002 alone vs. 3.82 ± 0.13 in LY-294002 plus Ang II, n = 3 experiments, *p* < 0.05). In addition, treatment with LY-294002 alone did not alter basal autophagy in cardiomyocytes. These results indicated that blockade of PI3KC1 significantly increased Ang II-induced autophagy in cardiomyocytes, suggesting that PI3KC1 plays an inhibitory role in Ang II-induced autophagy.

### Role of PI3KC1 in Ang II-Induced ROS Production

**FIGURE 4:**
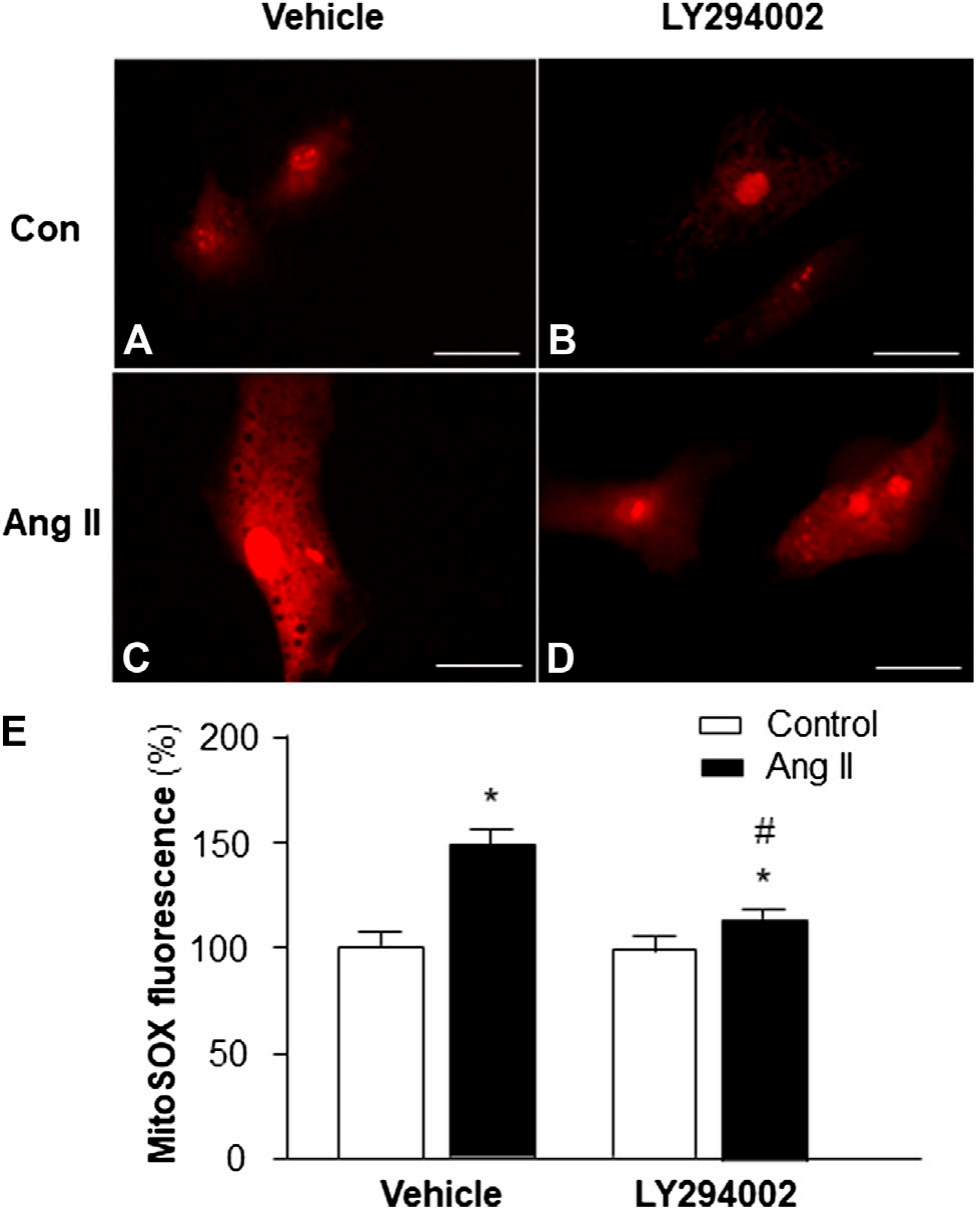
Effect of Ang II and blockade of PI3KC1 on mitochondrial ROS production in cardiomyocytes. Mitochondrial ROS levels were determined using the oxidant-sensitive fluorogenic probe, MitoSOX, in cardiomyocytes treated with vehicle control, Ang II (10^−6^ M) with or without the PI3C1 inhibitor, LY294002 (10^−6^ M). **(A–D)** Representative fluorescence micrographs of cultured cardiomyocytes probed by MitoSOX after treatments. **(E)** Bar graphs summarizing the mitochondrial ROS levels in cardiomyocytes treated with the conditions described above. The scale in the images is 25 μm. Data are presented as means ± SE, which were derived from three experiments and at least triplicate wells in each experiment. *p < 0.01 vs. cardiomyocytes treated vehicle control. #*p* < 0.05 vs. cardiomyocytes treated with Ang II.

Based on the results in the experiment above, showing that PI3KC1 has an inhibitory effect on Ang II-induced autophagy, we can propose that PI3KC1-mediated inhibition of autophagy could cause accumulation of damaged mitochondria, leading to elevation in intracellular ROS levels. Therefore, we next determined the role of PI3KC1 in Ang II-induced mitochondrial ROS production. Mitochondrial ROS production was measured using the MitoSox fluorescence approach in cardiomyocytes treated by control or Ang II (10^−6^ M) with or without the PI3KC1 inhibitor, LY-294002 (10^−6^ M). The results are presented in Figure 4, demonstrating that treatment of cardiomyocytes with Ang II significantly increased mitochondrial ROS production in the presence of vehicle (DMSO, 0.1%) as expected (100 ± 2.4% in control vs 149.3 ± 3.6% in Ang II, n = 3 experiments, *p* < 0.01). More interestingly, treatment with LY-294002 dramatically attenuated Ang II-induced increases in mitochondrial ROS accumulation by 31.7% in cardiomyocytes (95.3 ± 2.2% in LY-294002 alone vs. 115.6 ± 3.0% in LY-294002 plus Ang II, n = 3 experiments, *p* < 0.05). In addition, treatment with LY-294002 alone did not alter basal mitochondrial ROS production. These results suggest that PI3KC1-induced inhibition of autophagy may contribute to the Ang II treatment-associated elevation in intracellular ROS levels in cardiomyocytes.

### Role of PI3KC1 in Ang II-Induced Cardiac Hypertrophy

**FIGURE 5:**
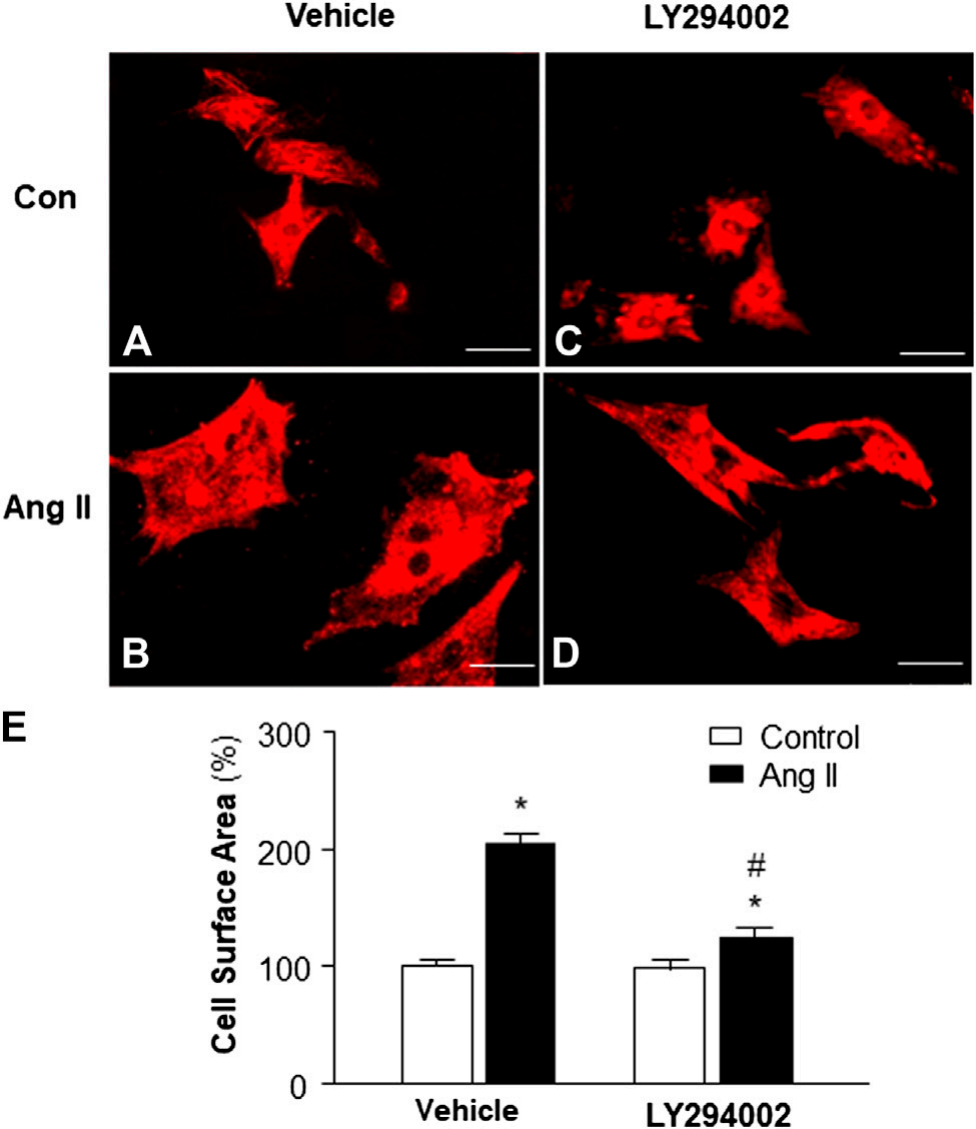
Effect of Ang II and blockade of PI3KC1 on cardiomyocyte hypertrophy. Morphology of cardiomyocytes were determined by immunocytochemistry using an α-sarcomeric actin antibody in cardiomyocytes after treated with vehicle control, Ang II (10^−6^ M) with or without the PI3KC1 inhibitor, LY294002 (10^−6^ M) for 24 h **(A–D)** Representative fluorescence micrographs of cultured cardiomyocytes stained with α-sarcomeric actin antibody after treatments. **(E)** Bar graph summarizing the size of cardiomyocytes after the indicated treatment. Data are means ± SE, derived from three experiments and at least triplicate wells in each experiment. *p < 0.05 vs. control; #*p* < 0.05 vs. Ang II treatment.

Increasing evidence indicates that elevated mitochondrial ROS is involved in Ang II-induced cardiac hypertrophy (Guo et al., 2017). Considering the result from the experiment above, showing that PI3KC1 contributes to Ang II-induced elevation in intracellular ROS levels, we thus examined the role of PI3KC1 in Ang II-induced cardiac hypertrophy. The cell surface area was measured under a fluorescence microscope in cardiomyocytes stained with α-sarcomeric actin antibodies after treatment with control or Ang II (10^−6^ M) with or without a PI3KC1 inhibitor, LY-294002 (10^−6^ M). The results are presented in Figure 5, showing that treatment with Ang II significantly increased the cell surface area by 2-fold and that co-treatment with LY294002 significantly attenuated the Ang II-induced increase in cell surface area. In addition, LY2940002 alone did not alter the basal size of the cells. These results demonstrate that blockade of PI3KC1 significantly attenuated Ang II-induced cardiac hypertrophy, suggesting that activation of PI3KC1 contributes to Ang II-induced cardiac hypertrophy.

### Role of PI3KC3 in Ang II-Induced Cardiac Hypertrophy

**FIGURE 6:**
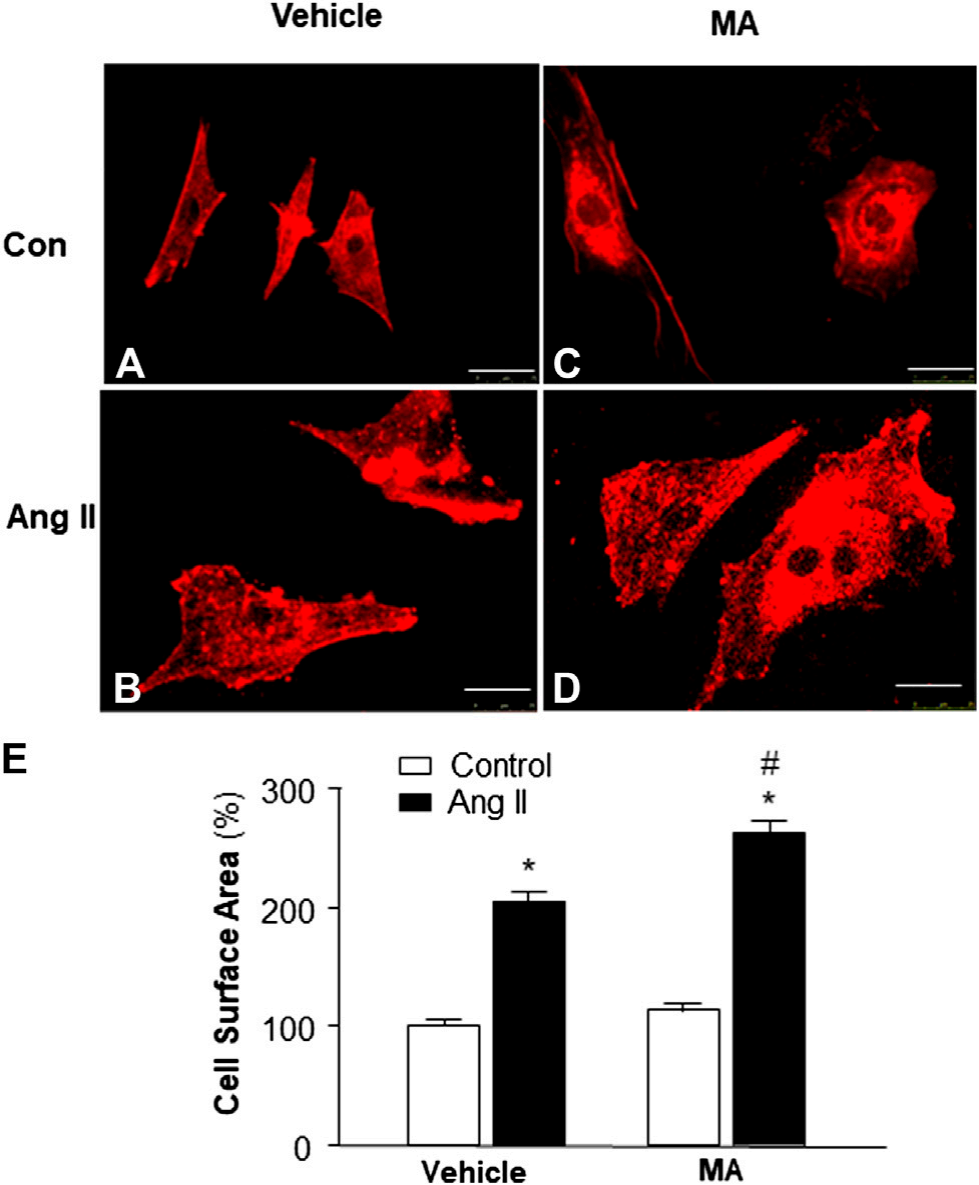
Effect of Ang II and blockade of PI3KC3 on cardiomyocyte hypertrophy. Cell morphology of cardiomyocytes was determined by immunocytochemistry using an α-sarcomeric actin antibody in cardiomyocytes after treatment with vehicle control, Ang II (10^−6^ M) with or without the PI3KC3 inhibitor, 3-methyladenine (3-MA, 10^−6^ M) for 24 h **(A–D)** Representative fluorescence micrographs of cultured cardiomyocytes stained with α-sarcomeric actin antibodies after treatments. (**E)** Bar graph summarizing the size of cardiomyocytes after the indicated treatment. Data are means ± SE, which were derived from three experiments and at least triplicate wells in each experiment. *p < 0.05 vs. control; #*p* < 0.05 vs. Ang II treatment.

Previous studies demonstrate that mice with a PI3KC3 gene deletion have increased heart size (Jaber et al., 2012). Thus, we examined the role of PI3KC3 in Ang II-induced cardiac hypertrophy. The size of cardiomyocytes was measured under a fluorescence microscope in cardiomyocytes stained with α-sarcomeric actin antibodies after treatment with control or Ang II (10^−6^ M) with or without a PI3KC3 inhibitor, 3-methyladenine (3-MA, 10^−6^ M). The results are presented in Figure 6, demonstrating that treatment of cardiomyocytes with Ang II significantly increased the cell surface area as expected. More interestingly, co-treatment with 3-MA significantly facilitated Ang II-induced increases in cell surface area. Treatment with 3-MA alone did not significantly alter the basal size of the cells. These results demonstrate that blockade of PI3KC3 significantly promotes Ang II-induced cardiac hypertrophy, suggesting that PI3KC3 activation may have a protective effect on Ang II-induced cardiac hypertrophy.

### Role of PI3KC3 in Ang II-Induced ROS Production

**FIGURE 7:**
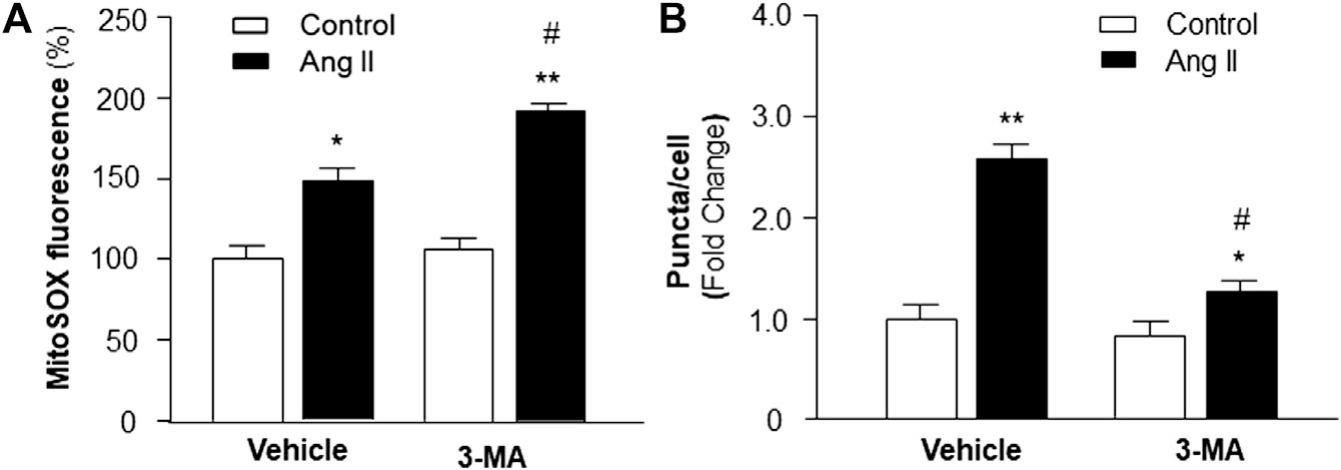
Effect of Ang II and blockade of PI3KC3 on mitochondrial ROS production and autophagy in cardiomyocytes. Mitochondrial ROS levels were determined using the oxidant-sensitive fluorogenic probe, MitoSOX and autophagy was examined using MAP-LC3 antibodies in cardiomyocytes treated with vehicle control, Ang II with or without the PI3KC3 inhibitor, 3-methyladenine (3-MA). **(A)** Bar graphs summarizing the mitochondrial ROS levels in cardiomyocytes treated with vehicle control, Ang II (10^−6^ M), 3-MA (10^−6^ M), and Ang II + 3-MA. Data are presented as means ± SE, which were derived from three experiments and at least triplicate wells in each experiment. *p < 0.05 and **p < 0.01 vs. cardiomyocytes treated vehicle control. #*p* < 0.05 vs. cardiomyocytes treated with Ang II. **(B)** Bar graphs summarizing quantitative analysis of autophagic alterations in cardiomyocytes treated with vehicle control, Ang II (10^−6^ M), 3-MA (10^−6^ M), and Ang II + 3-MA. Data are means ± SE, which were derived from three experiments and at least triplicate wells in each experiment. *p < 0.05 and **p < 0.01 vs. cardiomyocytes that treated with vehicle control. #*p* < 0.05 vs. cardiomyocytes that treated with Ang II.

Previous studies demonstrate that increased intracellular ROS production contributes to cardiac hypertrophy (Sag et al., 2014); thus, we examined the hypothesis that PI3KC3 reduces intracellular ROS production, leading to a protective effect on Ang II-induced cardiac hypertrophy. Mitochondrial ROS levels were detected using MitoSOX staining in cardiomyocytes after treatment with control or Ang II (10^−6^ M) with or without PI3KC3 inhibitor, 3-MA (10^−6^ M). The results are presented in Figure 7A, demonstrating that Ang II treatment significantly increased MitoSOX fluorescence density, suggesting elevated ROS levels, as expected. More interestingly, co-incubation with 3-MA significantly facilitated Ang II-induced increases in mitochondrial ROS production. However, treatment with 3-MA alone did not significantly alter basal ROS production. These results demonstrate that blockade of PI3KC3 promoted Ang II-induced mitochondrial ROS production, suggesting that activation of PI3KC3 may exert a protective effect on Ang II-induced ROS generation in cardiomyocytes.

### Role of PI3KC3 in Ang II-Induced Autophagy

We next examined whether the protective effect of PI3KC3 on Ang II-induced ROS production is mediated by facilitating autophagy, leading to increased scavenging of damaged mitochondria, which is a major source of intracellular ROS. Autophagy was detected with MAP-LC3 antibody immunofluorescence by counting intracellular puncta as formation of autophagesomes in cardiomyocytes treated with control or Ang II (10^−6^ M) with or without 3-MA (10^−6^ M), a PI3KC3 inhibitor. The results are presented in Figure 7B, demonstrating that treatment of cardiomyocytes with Ang II significantly increased autophagy as compared with control. Co-treatment with 3-MA significantly attenuated Ang II-induced increases in autophagy. Treatment with 3-MA alone did not significantly alter the basal autophagy levels. These results demonstrate that blockade of PI3KC3 significantly attenuated Ang II-induced increases in autophagy, suggesting that activation of PI3KC3 contributes to Ang II-induced autophagy, facilitating salvage of damaged mitochondria, and thereby leading to reduced ROS production in cardiomyocytes.

## Discussion

**FIGURE 8:**
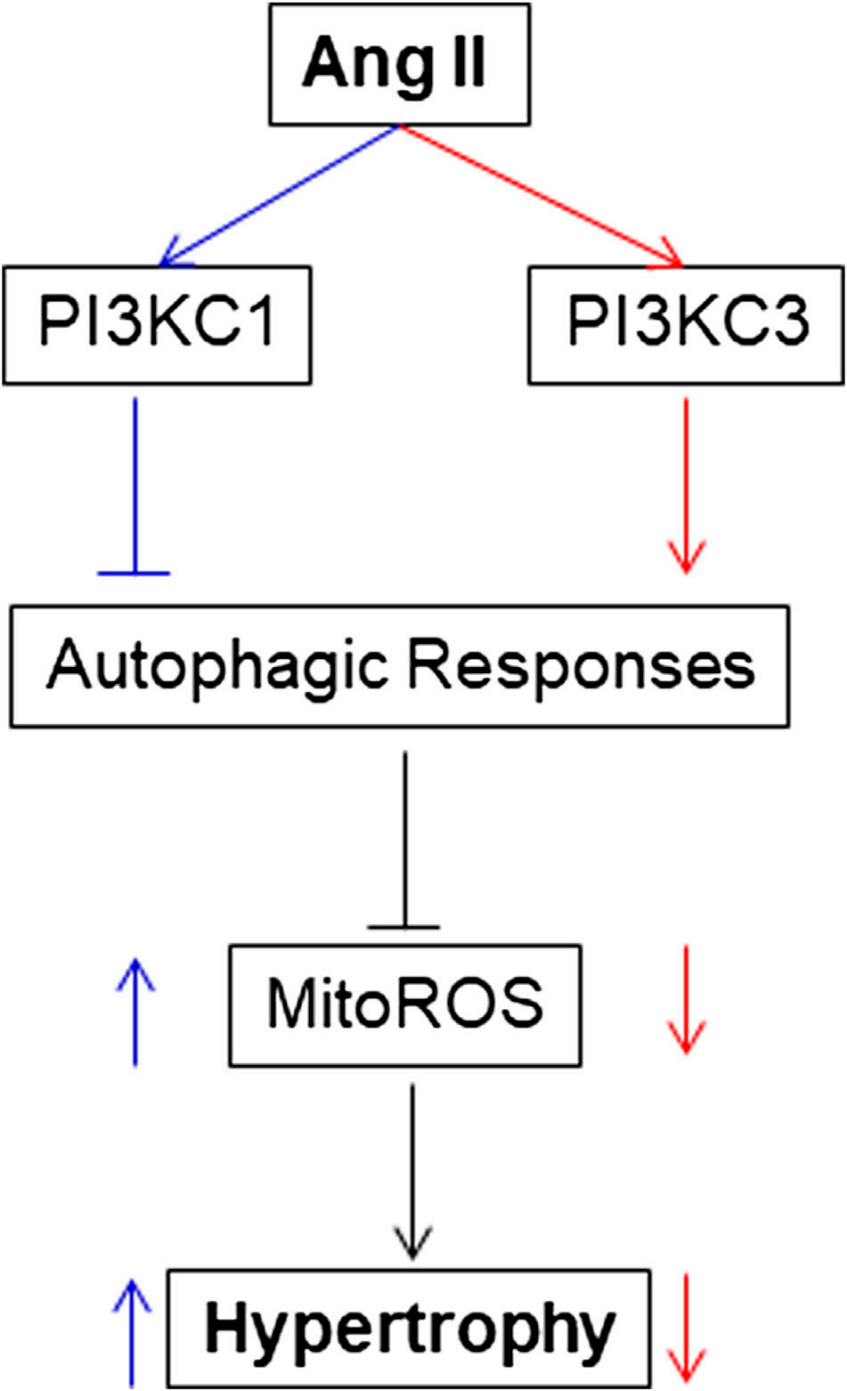
Proposed intracellular mechanisms involved in the action of PI3KC1 vs. PI3KC3 in the Ang II-induced cardiac hypertrophy. PI3KC1, PI3-kinase Class I; PI3KC3, PI3-kinase Class III; ROS, reactive oxygen species; MitoROS, mitochondrial ROS.

The present study provides the first evidence that PI3KC1 contributes to Ang II-induced cardiac hypertrophy by inhibiting autophagy and increasing mitochondrial ROS production, and that PI3KC3 has a protective effect on Ang II-induced cardiac hypertrophy by promoting autophagy-mediated scavenging of mitochondrial ROS in cardiomyocytes (as summarized in Figure 8). This conclusion is supported by the following evidence: 1) blockade of PI3KC1 promotes Ang II-induced autophagy; in contrast, blockade of PI3KC3 attenuates Ang II-induced autophagy; 2) blockade of PI3KC1 diminishes Ang II-induced increases in mitochondrial ROS production and hypertrophy; 3) blockade of PI3KC3 promotes the Ang II-induced increase in mitochondrial ROS production and hypertrophy; and 4) inhibition of PI3KC1 by viral vector-mediated over-expression of the dominant negative p85 subunit significantly attenuated Ang II-induced cardiac hypertrophy in the heart. Collectively, these findings are consistent with the idea that Class I and Class II PI3-kinases play a different role in Ang II-induced cardiac hypertrophy by regulating autophagy-mediated scavenging of mitochondrial ROS generation.

Previous studies have demonstrated that increased production of ROS is involved tou in Ang II-induced cardiac hypertrophy. It has been proposed that NADPH oxidase is the major source of Ang II-stimulated ROS generation in cardiomyocytes. This hypothesis is supported by a previous study showing that blockade of gp91^phox^, a subunit of NADPH oxidase, attenuates Ang II infusion-associated-hypertension and cardiac hypertrophy (Bendall et al., 2002). However, blockade of gp91^phox^ associated ROS production has no preventive effect on cardiac hypertrophy in Ang II over-production mice with transgenic overexpression of renin (Touyz et al., 2005). In this regard, mitochondrial dysfunction has been proposed as another source of ROS. It has been reported that ROS derived from damaged mitochondria are involved in cardiac hypertrophy induced by hypertension and myocardial infarction (Pei et al., 2016). This hypothesis is supported by the results from the present study, showing that Ang II treatment significantly increased mitochondrial ROS generation in cardiomyocytes. Furthermore, increased mitochondrial ROS generation was associated with impaired autophagy, a vital intracellular scavenging mechanism for damaged mitochondria that generate ROS. More interestingly, this impaired autophagy induced by Ang II was attenuated by blockade of PI3KC1 in cardiomyocytes. Thus, inhibition of the PI3KC1 signaling pathway could generate a protective effect on cardiac hypertrophy. This hypothesis is validated in our study, which demonstrates that inhibition of PI3KC1 by viral vector-mediated overexpression of dominant negative p85 PI3KC1 subunit (Lv-DNp85) significantly attenuated Ang II perfusion-induced cardiac hypertrophy in rats. These results suggest that the PI3KC1 signaling pathway could be a potential target for the treatment or prevention of cardiac hypertrophy.

One major question that arises from our study concerns the signaling pathways involved in the inhibitory effect of PI3KC1 on autophagy. It has been shown that PI3KC1 phosphorylates protein kinase B/Akt and that Akt induces activation of mTOR, which regulates several cellular functions such as cell growth, proliferation, and autophagy (Vanhaesebroeck et al., 2012; Sciarretta et al., 2018). This observation is consistent with the results from the current study showing that blockade of PI3KC1 with Lv-DNp85 significantly attenuated Ang II-induced phosphorylation of Akt in the heart of rats. In the heart, pharmacological inhibition of mTOR with rapamycin reverses cardiac hypertrophy induced by Akt overexpression (Shioi et al., 2002; Shiojima et al., 2015). In addition, it has been reported that activation of the mTOR signaling pathway inhibits autophagy activity in the heart (Shiojima et al., 2015; Nakai et al., 2007). Therefore, we can propose that the Akt/mTOR signaling pathway could be involved in the PI3KC1 activation-associated impairment of autophagy in cardiomyocytes. This hypothesis needs be verified in future studies.

This study demonstrates, for the first time, that the protective effects of PI3KC3 on Ang II-induced cardiac hypertrophy and mitochondrial ROS production are mediated by promoting autophagy activity. The autophagy mechanism includes the formation of double membraned autophagosomes, the fusion of autophagosomes to late endosomes/lysosomes, and the digestion of the enclosed contents by lysosomal hydrolases. Autophagy functions as an intracellular scavenging procedure by eliminating misfolded proteins and damaged organelles, such as damaged mitochondria, in order to maintain cellular homeostasis. The damaged mitochondria could increase ROS generation, leading to cardiac hypertrophy and heart failure (Bao et al., 2007). It has been reported that impaired autophagy is involved in the development of cardiac hypertrophy (Wang and Cui, 2017). In the current study, we observed that blockade of PI3KC3 significantly inhibited Ang II-induced autophagy activity, leading to increased ROS generation and cardiomyocyte hypertrophy. These findings suggest that PI3KC3 has a protective role in Ang II-induced cardiac hypertrophy by suppression of mitochondrial ROS via up-regulation of autophagy activity. This conclusion is supported by a previous investigation, from Jaber et al. (Jaber et al., 2012), demonstrating that mice with ablation of PI3KC3 Vps34 develop severe cardiac hypertrophy and reduced heart contractility. However, this conclusion will require verification in future studies using animal models.

In summary, we provide the first evidence that both PI3KC1 and PI3KC3 are involved in Ang II-induced cardiac hypertrophy, with each kinase playing a different role: Activation of PI3KC1 negatively regulates autophagy activity, leading to increased mitochondrial ROS production and cardiac hypertrophy. In contrast, activation of PI3KC3 increases autophagy activity, leading to reduced mitochondrial ROS generation and a protective effect on Ang II-induced cardiac hypertrophy. An interesting question that arose from the current studies is why Ang II treatment causes autophagic alterations with two phases: an increasing phase at lower dosages and a decreasing phase at high dosages. The possible underlying mechanism may be due to activation of different PI3Ks. At lower dosage, Ang II predominantly activates PI3KC3, leading to increases in autophagic responses and a protective effect on mitochondrial ROS accumulation. This protective effect may contribute to the compensatory cardiac hypertrophy effect under physiological conditions. It will be very interesting to study whether this protective signaling pathway induced by Ang II at lower dosages is mediated by stimulation of AT2 receptors since activation of AT2 receptors is reported to exhibit a protective effect from cardiac hypertrophy (Castoldi et al., 2019). At higher dosages, Ang II may predominantly activates PI3KC1, besides PI3KC3, leading to inhibition of autophagic responses and accumulation of mitochondrial ROS, which results in a detrimental effect and hypertrophy in the heart. However, this hypothesis needs further investigation in the future. Taken together, the results indicate that the shift from PI3KC3 to PI3KC1 may be involved in the conversion from cardiac compensation to decompensation in pathophysiological conditions; therefore, PI3KC1 and PI3KC3 may be novel therapeutic targets for the prevention or treatment of cardiac hypertrophy.

## Data Availability

The raw data supporting the conclusions of this article will be made available by the authors, without undue reservation.
